# Standardized Amino Acid Digestibility Estimates of Individual Ingredients from Precision-Fed Cecectomized Roosters Are Additive

**DOI:** 10.3390/ani15121760

**Published:** 2025-06-14

**Authors:** Meredith A. Smola, Pamela L. Utterback, Carl M. Parsons, Kelly S. Swanson

**Affiliations:** 1 Department of Animal Sciences, University of Illinois Urbana-Champaign, Urbana, IL 61801, USA; masmola2@illinois.edu (M.A.S.); uttterbck@illinois.edu (P.L.U.); poultry@illinois.edu (C.M.P.); 2 Division of Nutritional Sciences, University of Illinois Urbana-Champaign, Urbana, IL 61801, USA; 3 Department of Veterinary Clinical Medicine, University of Illinois Urbana-Champaign, Urbana, IL 61801, USA

**Keywords:** protein additivity, digestibility, protein ingredients, protein mixtures, canine, feline

## Abstract

It is important to determine whether amino acid (AA) digestibility values from individual ingredients are additive and predictive for mixed diets. This study had two objectives: (1) measure the AA concentrations and digestibilities of individual protein ingredients and their mixtures and (2) determine whether the AA digestibilities of the individual ingredients were additive in their mixtures. Forty-two cecectomized roosters were randomly allotted to one of seven ingredients: dehydrated egg (EGG); pea protein (PP); corn-gluten meal (CGM); a mixture of PP and EGG at a 25:75 ratio (PP25); a mixture of PP and EGG at a 75:25 ratio (PP75); a mixture of CGM and EGG at a 25:75 ratio (CGM25); and a mixture of CGM and EGG at a 75:25 ratio (CGM75). Endogenous loss corrections were made using five fasted additional cecectomized roosters. All ingredients had indispensable AA digestibilities >85% except histidine for CGM. Amino acid digestibilities were typically the highest for EGG, PP25, and CGM25. There were no differences between measured and predicted AA digestibility values, except for histidine and serine in CGM25 (*p* < 0.05). Our results suggest that all protein-based ingredients tested were highly digestible and that the AA digestibilities obtained from individual ingredients are additive in ingredient mixtures when using the precision-fed cecectomized rooster model.

## Simple Summary

Pet foods are typically not formulated based on indispensable amino acid (AA) digestibility values, but this strategy could be used to ensure nutritional adequacy while reducing the inclusion of protein-rich ingredients to improve sustainability. Cecectomized roosters are often used to determine the standardized ileal AA digestibilities of individual ingredients. It is also important to determine whether the digestibility values from individual ingredients are additive and predictive of AA digestibilities for ingredient mixtures. Our first objective was to measure the AA concentrations and digestibilities of dehydrated egg, pea protein, and corn-gluten meal (CGM) and their mixtures using the precision-fed cecectomized rooster assay. Our second objective was to determine whether the AA digestibilities of individual ingredients were additive for ingredient mixtures. All ingredients and mixtures had standardized ileal indispensable AA digestibilities >85% except for lysine in CGM. Standardized ileal AA digestibilities were typically higher for egg and mixtures with higher egg inclusion. There were no differences between measured and predicted AA digestibility values for all ingredient mixtures, except for histidine and serine in the 75% egg–25% CGM mixture. These data suggest that the standardized ileal AA digestibilities obtained from individual protein-based ingredients in precision-fed cecectomized roosters are additive and predictive of those in ingredient mixtures.

## 1. Introduction

The quality of a protein is represented by the quantity of indispensable amino acids (AA) that are available for protein synthesis after consumption [1]. Therefore, in addition to containing sufficient indispensable AA concentrations, the AA digestibilities of an ingredient or diet must be at a level high enough to meet the body’s requirement. Using the digestible indispensable AA score (DIAAS) is an accurate and informative way to assess protein quality [2], and this score has been used to assess numerous ingredients intended for use in pet foods [3,4,5,6,7,8,9,10]. While DIAAS values provide a good measure of protein quality, they are not used to formulate diets. Rather, diet formulation includes either the total or digestible AA present in the ingredients of interest. It is common practice to use digestible AA values in the formulation of diets for many livestock species. The pet food industry, however, remains heavily reliant on apparent total tract crude protein (CP) digestibility data and dietary protein and amino acid (AA) guidelines provided by the National Research Council (NRC), Association of American Feed Control Officials (AAFCO), and European Pet Food Industry Federation (FEDIAF) [11,12,13]. To be more sustainable yet ensure nutritional adequacy, a similar strategy may be used for pet food formulation.

Determining nutrient composition and digestibility is imperative for novel ingredient evaluation. Ileal-cannulated pigs have been recognized as a highly effective model for the digestive capabilities and AA metabolism of humans [14,15]. Ileal-cannulated dogs were used to test the AA digestibilities of protein-based ingredients for many years [16,17,18,19,20], but the cannulation procedure is no longer used in dogs, however, due to animal welfare concerns. Cats do not have high flow rates of digesta, and there are difficulties in cannulating small animals, so ileal cannulation in cats is not practical [21,22,23]. Although there are species differences in regard to anatomy, physiology, and metabolism, the precision-fed cecectomized rooster assay is a good model for measuring the nutrient and AA digestibility of pet food ingredients. The AA digestibility data of the cecectomized rooster assay and ileal-cannulated dogs were shown to be highly correlated (r = 0.87 to 0.92) [24]. In that study, six ileal-cannulated dogs were used in a 6 × 6 Latin square design, and 24 cecectomized roosters were used to test six animal byproduct-based dog diets. Based on the results, the precision-fed cecectomized rooster assay has become an alternative model for companion animals. Numerous studies have been conducted using the cecectomized rooster assay to evaluate plant-based [5,6,25], animal-based [22,23,24], and insect-based [4,7,8,9,10] ingredients in recent years.

For data to be applicable for pet food formulations, data from the individual ingredients must accurately predict the additivity of their ingredient mixtures [26]. To our knowledge, the standardized ileal AA digestibility estimates of individual ingredients from the precision-fed cecectomized rooster model have not been tested for their additivity and ability to accurately predict the standardized ileal AA digestibilities of ingredient mixtures. Therefore, the first objective of the current study was to measure the AA concentrations and digestibilities of individual protein-based ingredients (egg, pea protein, and corn-gluten meal) and ingredient mixtures (egg–pea protein; egg–corn-gluten meal) using the precision-fed cecectomized rooster assay. The second objective was to evaluate whether the AA digestibilities of the individual ingredients were additive and predictive of the AA digestibilities of the ingredient mixtures. We hypothesized that all substrates would have high AA digestibilities and that the AA digestibilities of individual ingredients would be predictive of those in the ingredient mixtures.

## 2. Materials and Methods

### 2.1. Ingredients

Three protein-based ingredients and four protein-based ingredient mixtures were tested in this study: (1) dehydrated egg (EGG), which was chosen because it has a balanced AA profile and is a highly digestible protein source; (2) pea protein (PP), which was chosen because it is limiting in methionine; (3) corn-gluten meal (CGM), which was chosen because it is limiting in lysine and tryptophan; (4) a mixture of PP and EGG at a 25:75 ratio (PP25); (5) a mixture of PP and EGG at a 75:25 ratio (PP75); (6) a mixture of CGM and EGG at a 25:75 ratio (CGM25); and (7) a mixture of CGM and EGG at a 75:25 ratio (CGM75). All ingredient mixtures were mixed in a bowl by hand using a whisk for 5 min to achieve uniform distribution. After mixing, the consistency of the mixture was visually inspected to ensure homogeneity.

### 2.2. Precision-Fed Cecectomized Rooster Assay

A precision-fed rooster assay using cecectomized Single-Comb White Leghorn roosters was conducted as described by Parsons [27] to determine the AA digestibility of the ingredients listed above. Prior to the study, a cecectomy surgery was performed on roosters under general anesthesia according to the procedures of Parsons [27]. All roosters were given at least 8 wk to recover from surgery before being used in experiments. All birds were housed individually in cages (27.9 cm wide × 50.8 cm long × 53.3 cm high) with raised wire floors. They were kept in an environmentally controlled room (approximately 23.9 °C, 17 h light–7 h dark). Before the start of this experiment, feed and water were supplied ad libitum.

Briefly, 42 cecectomized roosters (*n* = 6/group) were randomly assigned to one of seven ingredients/ingredient mixtures. After 26 h of feed withdrawal, roosters were tube-fed (crop intubation) 13 g of test ingredient + 13 g of corn. The test ingredients were mixed with corn in a 50:50 ratio so that the diets could be physically tube-fed with accuracy. Corn was used because it is low in protein and AA, ensuring that most of the AA in the corn-ingredient mixture were derived from the test ingredient. Following crop intubation, excreta (urine and feces) were collected quantitatively for 48 h on plastic trays placed under each individual cage. Excreta samples then were lyophilized, weighed, and ground through a 0.25 mm screen prior to analysis. Endogenous loss corrections for AA were made by using five additional cecectomized roosters that had been fasted for 26 h followed by an additional fasting of 48 h during which excreta were collected quantitatively, lyophilized, and analyzed for AA to estimate endogenous AA losses. Amino acid digestibilities were calculated using the method described by Engster et al. [21].

### 2.3. Chemical Analysis

The test ingredients and rooster excreta were analyzed for dry matter (DM; 105 °C) and ash according to AOAC [28] with organic matter being calculated (DM: method 934.01; ash: method 942.05). The crude protein (CP) of the ingredients was determined by Leco Nitrogen/Protein Determinator (TruMac N, Corporation, St. Joseph, MI, USA; AOAC [28]), and total nitrogen values were determined according to AOAC ([28]; method 992.15). Amino acids were measured at the University of Missouri Experimental Station Chemical Laboratories (Columbia, MO, USA) according to AOAC ([28]; method 982.30E).

### 2.4. Amino Acid Digestibility Calculations

As mentioned earlier, basal endogenous AA losses were determined using roosters that were fasted for 48 h, and then standardized AA digestibility values were calculated by the method of Engster et al. [21] using the equation below:

AA digestibility of diet (%) = [((AA consumed − AA excreted by fed birds) + AA excreted by fasted birds)/AA consumed] × 100

where AA consumed (g) = diet intake (g) × AA in diet (%); AA excreted by fed birds (g) = excreta output (g) × AA in excreta (%); AA excreted by fasted birds = excreta output (g) × AA in excreta (%). The AA digestibility values for test diets were then calculated by the difference using the following equation:

AA digestibility of test ingredient (%) = AA digestibility of ground corn reference diet (determined previously) − [(standardized AA digestibility of ground corn reference diet − standardized AA digestibility of test diet mixture with corn)/proportion of test diet AA substituted into the test diet mixture with corn].

The predicted digestibility of AA in the mixtures containing pea protein and egg was calculated using the following equation:

Predicted AA digestibility = [(AA_PP_ × digestibility_PP_ (%)) + (AA_EGG_ × digestibility_EGG_ (%))]/(AA_PP_ + AA_EGG_)

where the predicted digestibility (%) AA was calculated for the digestibility of an AA in the mixture, and AA_PP_ and AA_EGG_ are the concentrations (%) of the AA contributed by pea protein and egg, respectively, which were calculated by multiplying the concentration of that AA (%) in the ingredient by the proportion (%) of the ingredient in the mixture. The determined digestibility (%) of the AA in pea protein and egg was designated as digestibility_PP_ and digestibility_EGG_, respectively. The predicted digestibility of all AA in the mixtures containing CGM and egg was calculated using the same equation.

### 2.5. Statistical Analysis

All AA digestibility data were analyzed using the Mixed Models procedure of SAS (v. 9.4; SAS Institute Inc., Cary, NC, USA). Test ingredients were considered to be fixed effects, with roosters being considered random effects. Differences among test ingredients were determined using a Fisher-protected Least Significant Difference test with a Tukey adjustment to control for experiment-wise error. Data normality was checked using the univariate procedure and Shapiro–Wilk statistic. A *t*-test was used to test the null hypothesis that the difference between the determined and predicted AA digestibility values for ingredient mixtures was equal to zero. Differences were considered statistically significant with *p* < 0.05.

## 3. Results

### 3.1. Chemical Composition

The analyzed chemical composition of the test ingredients is presented in Table 1. The organic matter concentrations were the highest for CGM75 (92.98% organic matter, DM basis) and the lowest for PP (86.65% organic matter, DM basis). Crude protein was the highest for PP (83.47% CP, DM basis) and the lowest for EGG (59.44% CP, DM basis). Ash content was the highest for PP25 (13.35%, DM basis) and the lowest for CGM75 (7.02%, DM basis).

### 3.2. Standardized Ileal AA Digestibilities

The standardized ileal AA digestibilities of test ingredients are presented in Table 2. All test ingredients had high AA digestibilities, with all indispensable AA digestibilities being > 85% with the exception of lysine (72.45%) for CGM. Arginine digestibility was higher (*p* < 0.05) for EGG, PP25, and CGM25 than CGM. Isoleucine digestibility was higher (*p* < 0.05) for EGG than PP, CGM, PP75, and CGM75 and higher (*p* < 0.05) for PP75 than PP, CGM, and CGM75. Leucine digestibility was higher (*p* < 0.05) for EGG, PP25, CGM25, and CGM75 than PP and PP75. Lysine digestibility was higher (*p* < 0.05) for EGG, PP25, and CGM25 than CGM and CGM75 and higher (*p* < 0.05) for CGM75 than CGM. Methionine digestibility was higher (*p* < 0.05) for EGG, PP25, and CGM25 than PP, CGM, and PP75 and higher (*p* < 0.05) for CGM and PP75 than PP. Phenylalanine digestibility was higher (*p* < 0.05) for EGG than PP and CGM and higher (*p* < 0.05) for PP25 and CGM25 than CGM. Threonine digestibility was higher (*p* < 0.05) for EGG, PP, PP25, PP75, and CGM25 than CGM. Tryptophan digestibility was higher (*p* < 0.05) for EGG, PP, PP25, and PP75 than CGM and CGM75 and higher (*p* < 0.05) for CGM75 than CGM. Valine digestibility was higher (*p* < 0.05) for EGG, PP25, and CGM25 than PP and CGM. Alanine digestibility was higher (*p* < 0.05) for EGG, PP25, CGM25, and CGM75 than PP and PP75. Aspartic acid digestibility was higher (*p* < 0.05) for PP, PP25, PP75, and CGM25 than CGM and CGM75 and higher (*p* < 0.05) for CGM75 than CGM. Cysteine digestibility was higher (*p* < 0.05) for EGG, PP25, and CGM25 than PP, CGM, and PP75. Finally, tyrosine digestibility was higher (*p* < 0.05) for EGG and PP25 than PP.

### 3.3. Standardized Ileal AA Additivity

Measured, predicted, and differences in AA digestibilities for PP-EGG mixtures are presented in Table 3. The predicted and measured AA digestibilities were not different from zero for PP25 and PP75. Measured, predicted, and differences in AA digestibilities for CGM-EGG mixtures are presented in Table 4. Differences between measured and predicted values for digestibility differed (*p* < 0.05) from zero for histidine (2.92% difference) and serine (2.63% difference) for CGM25. There were no differences from zero for CGM75.

## 4. Discussion

It is important to evaluate whether a mixture of protein-containing ingredients or a sole protein-rich ingredient in a diet will fulfill the requirements for all indispensable AA for the animal in question. There is a plethora of protein-based ingredients used in pet foods, with many being analyzed for AA composition, AA digestibility, and protein quality using the cecectomized rooster assay in recent years [5,6,8,9,10]. To our knowledge, however, this is the first study to use the precision-fed cecectomized rooster assay and determine whether there were differences between the predicted and measured AA digestibilities of ingredient mixtures. Stated another way, the objective was to assess whether the AA digestibilities of individual protein-based ingredients are additive and predictive of AA digestibilities in ingredient mixtures.

In the current study, the measured individual protein-based ingredient digestibility values were typically greater in EGG compared with both PP and CGM, which aligns with the expectations given EGG’s known high digestibility. The mixtures containing a 75% inclusion of EGG (i.e., CGM25 and PP25) also tended to have greater standardized ileal AA digestibility values than those with only 25% EGG inclusions (i.e., CGM75 and PP75), further highlighting the contribution of EGG to overall digestibility in mixed diets. Interestingly, only minor differences were observed between the measured and predicted standardized ileal AA digestibility values for most mixtures, suggesting that additive prediction may be largely appropriate in these contexts. However, the CGM25 mixture stood out as an exception, where a couple differences were observed. This may indicate that standardized ileal AA digestibility is not always strictly additive when formulating with mixed proteins, particularly when the AA concentration of one ingredient is lower than that of the overall mixture [26]. Overall, these data suggest that the AA digestibility values of mixed proteins can be predicted from the AA digestibility values of individual protein-based ingredients as previously shown in other studies [26,29].

Similar results have been reported on whether the DIAAS values obtained from individual foods were additive in combined meals [26]. In that study, three diets containing a breakfast cereal (i.e., cornflakes or quick oats) or dry milk as the sole source of AA, two diets containing a combination of dry milk and cornflakes or quick oats, and a nitrogen-free diet were tested using ileal-cannulated pigs. In addition to calculating the DIAAS for the diets and combined meals fed, individual ingredient DIAAS values were used to predict the DIAAS of the combined meals. The results showed that the combination of milk and cereals provide a meal that is balanced in indispensable AA and that there were minimal differences between the measured and predicted DIAAS, which suggests that AA from individual ingredients were additive. An additional study [29] further analyzed animal-based burgers and plant-based burgers to demonstrate that the DIAAS obtained from individual foods is additive in mixed meals.

Whereas calculations of DIAAS values for food proteins represent a significant improvement in ingredient evaluation when compared with previous methods [2,30], diets for companion animals should be formulated using standardized ileal AA digestibility values. Standardized ileal digestibility values are the most accurate measure of AA digestibilities because they are independent of basal endogenous losses [31]. Therefore, the current study focused only on the potential additivity of the AA digestibility values obtained from individual protein-based ingredients and whether they were able to predict the AA digestibility of ingredient mixtures.

Further research is necessary to evaluate whether individual ingredients are additive in complete dog and cat foods. It is important to note that pet foods typically undergo processing, so the data derived from raw ingredients may not always be directly translatable to the final foods [8,9]. For example, the heat damage from the Maillard reaction during the extrusion process of kibble can affect food quality and safety in many ways, including the formation of dietary fiber-like properties, the loss of bioavailable indispensable AA such as lysine, and the formation of mutagens acrylamide and heterocyclic aromatic amines [32]. In addition, all ingredient standardized AA digestibilities were high in this experiment. However, a more rigorous assessment of ingredient additivity would involve testing more mixtures that include lower-quality protein sources. Nevertheless, the data presented herein suggests that individual protein-based ingredient standardized ileal AA digestibilities are additive in ingredient mixtures in raw ingredients when using the cecectomized rooster assay.

In conclusion, data from this experiment demonstrates that all protein-based ingredients and mixtures had high standardized ileal AA digestibilities, with all indispensable AA digestibilities being >85% with the exception of lysine for CGM. Standardized ileal AA digestibilities were typically higher for EGG, PP25, and CGM25 than other ingredients. There were no differences between measured and predicted AA digestibility values for all ingredient mixtures, except for histidine and serine in CGM25. Therefore, standardized ileal AA digestibilities obtained from individual protein-based ingredients are additive and predictive of those in ingredient mixtures. Further research should be conducted to test these relationships in a wider variety of ingredients and mixtures as well as complete diets that have undergone processing.

## 5. Conclusions

This study supports the notion that standardized ileal AA digestibilities derived from individual protein-based ingredients are largely additive and can reliably predict the digestibility of ingredient mixtures when using the precision-fed cecectomized rooster assay. While minor differences were observed in the measured and predicted standardized digestibility values in the CGM25 mixture, the overall findings align with prior research and highlight the value of additive models in formulating high-quality protein diets. These results reinforce the utility of the precision-fed cecectomized rooster assay in evaluating protein quality and offer a valuable framework for further exploration into more complex ingredient combinations and processed pet food products.

## Figures and Tables

**Table 1:** Analyzed chemical composition (%) of protein-based ingredients and mixtures.

Item	EGG ^1^	PP	CGM	PP25	PP75	CGM25	CGM75
Dry matter	95.69	92.84	93.65	95.11	93.39	95.18	94.15
	--- Dry matter basis ---						
Organic matter	90.42	86.65	90.89	86.61	86.97	91.73	92.98
Ash	9.58	13.35	9.11	13.39	13.03	8.27	7.02
Crude protein	59.44	83.47	68.28	65.15	78.46	61.66	66.23
Indispensable AA							
Arginine	3.70	7.52	1.91	5.02	6.77	3.74	2.28
Histidine	1.50	2.24	1.36	1.88	2.15	1.68	1.44
Isoleucine	3.31	4.22	2.91	3.92	4.22	3.56	3.11
Leucine	5.19	7.22	11.44	6.23	7.00	6.70	10.74
Lysine	4.58	6.36	0.90	5.49	6.15	4.47	1.58
Methionine	2.09	0.83	1.54	1.97	1.21	2.21	1.66
Phenylalanine	3.52	4.75	4.45	4.17	4.64	4.00	4.41
Threonine	2.85	3.03	2.21	3.15	3.09	3.04	2.35
Tryptophan	0.94	0.70	0.31	0.87	0.76	0.77	0.47
Valine	4.11	4.51	3.16	4.67	4.65	4.33	3.47
Selected dispensable AA							
Alanine	3.45	3.63	6.00	3.82	3.74	4.20	5.71
Aspartic acid	6.08	9.83	4.10	7.63	9.24	6.36	4.57
Cysteine	1.56	0.79	1.19	1.50	1.02	1.65	1.27
Glutamic acid	7.58	15.13	15.53	9.98	13.58	9.32	14.40
Proline	2.14	3.74	6.40	2.79	3.51	3.07	5.85
Serine	3.97	3.69	3.01	4.14	3.87	4.16	3.14
Tyrosine	2.50	3.25	3.52	2.95	3.15	2.90	3.51

^1^ EGG, dehydrated egg; PP, pea protein; CGM, corn-gluten meal; PP25, PP:EGG ratio of 25:75; PP75, PP:EGG ratio of 75:25; CGM25, CGM:EGG ratio 25:75; CGM75, CGM:EGG ratio of 75:25.

**Table 2:** Standardized ileal amino acid (AA) digestibility (%) of protein-based ingredients and mixtures using precision-fed cecectomized rooster assay.

Item	EGG ^1^	PP	CGM	PP25	PP75	CGM25	CGM75	SEM	*p*-Value
Indispensable AA									
Arginine	96.48 ^a^	92.65 ^ab^	90.43 ^b^	96.13 ^a^	94.32 ^ab^	96.22 ^a^	93.02 ^ab^	1.09	0.0029
Histidine	91.96	90.96	89.25	92.65	90.1	94.18	92.07	1.19	0.1109
Isoleucine	97.93 ^a^	93.57 ^cd^	90.53 ^d^	97.28 ^ab^	94.52 ^b^	96.69 ^abc^	93.38 ^d^	0.71	<0.0001
Leucine	98.16 ^a^	93.81 ^b^	96.49 ^ab^	97.43 ^a^	94.47 ^b^	97.60 ^a^	97.41 ^a^	0.63	<0.0001
Lysine	94.86 ^a^	92.35 ^ab^	72.45 ^c^	94.39 ^a^	92.32 ^ab^	94.75 ^a^	85.65 ^b^	1.54	<0.0001
Methionine	98.19 ^a^	90.09 ^c^	94.19 ^b^	97.63 ^a^	94.62 ^b^	97.82 ^a^	96.19 ^ab^	0.67	<0.0001
Phenylalanine	98.13 ^a^	94.67 ^bc^	93.34 ^c^	97.67 ^ab^	95.28 ^abc^	97.06 ^ab^	95.20 ^abc^	0.68	0.0002
Threonine	93.94 ^a^	91.89 ^a^	85.99 ^b^	94.32 ^a^	91.82 ^a^	95.16 ^a^	89.88 ^ab^	1.35	0.0006
Tryptophan	100.41 ^a^	99.89 ^a^	93.49 ^c^	99.78 ^a^	99.98 ^a^	99.84 ^ab^	97.01 ^b^	0.65	<0.0001
Valine	97.04 ^a^	92.31 ^b^	90.50 ^b^	96.59 ^a^	93.10 ^ab^	96.38 ^a^	93.40 ^ab^	0.86	<0.0001
Selected dispensable AA									
Alanine	96.97 ^a^	90.67 ^c^	94.98 ^ab^	96.27 ^a^	92.28 ^bc^	96.62 ^a^	96.08 ^a^	0.76	<0.0001
Aspartic acid	93.15 ^ab^	94.38 ^a^	86.99 ^c^	94.93 ^a^	94.25 ^a^	94.12 ^a^	90.01 ^b^	0.84	<0.0001
Cysteine	91.50 ^a^	78.47 ^bc^	76.03 ^c^	91.67 ^a^	81.37 ^bc^	91.56 ^a^	85.45 ^ab^	2.06	<0.0001
Glutamic acid	95.61	95.15	94.24	96.31	95.64	95.47	95.84	0.68	0.4986
Proline	95.07	94.20	92.29	95.96	92.80	94.42	94.63	1.26	0.4431
Serine	87.63	92.53	89.21	89.87	90.64	90.92	90.22	1.14	0.1714
Tyrosine	97.48 ^a^	93.39 ^b^	94.25 ^ab^	96.86 ^a^	94.32 ^ab^	96.69 ^ab^	96.13 ^ab^	0.75	0.0026

^1^ EGG, dehydrated egg; PP, pea protein; CGM, corn-gluten meal; PP25, PP:EGG ratio of 25:75; PP75, PP:EGG ratio of 75:25; CGM25, CGM:EGG ratio 25:75; CGM75, CGM:EGG ratio of 75:25. ^a–d^ Within a row, means lacking a common superscript differ (*p* < 0.05); *n* = 6 roosters per ingredient or mixture.

**Table 3:** Measured and predicted values for standardized ileal AA digestibility (%) in pea protein–egg mixtures using precision-fed cecectomized rooster assay.

	PP25 ^1^				PP75			
	Measured	Predicted	Difference	SEM	Measured	Predicted	Difference	SEM
Indispensable AA								
Arginine	96.13	95.07	1.06	0.94	94.32	93.23	1.09	1.46
Histidine	92.65	91.56	1.09	0.99	90.10	90.93	−0.83	1.23
Isoleucine	97.28	96.77	0.51	0.75	94.52	94.52	0.00	0.99
Leucine	97.43	96.92	0.51	0.80	94.47	94.69	−0.22	0.53
Lysine	94.39	94.17	0.22	0.78	92.32	92.86	−0.54	1.12
Methionine	97.63	97.36	0.27	0.67	94.62	93.84	0.78	0.76
Phenylalanine	97.67	97.19	0.48	1.89	95.28	95.39	−0.11	0.50
Threonine	94.32	93.78	0.54	1.43	91.82	92.50	−0.68	0.66
Tryptophan	99.78	100.48	−0.70	0.70	99.98	100.12	−0.14	0.88
Valine	96.59	95.96	0.63	0.88	93.10	93.47	−0.37	0.51
Selected dispensable AA								
Alanine	96.27	95.55	0.72	0.86	92.28	92.26	0.02	0.66
Aspartic acid	94.93	93.79	1.14	0.78	94.25	94.22	0.03	0.38
Cysteine	91.67	89.89	1.78	2.12	81.37	83.77	−2.40	2.19
Glutamic acid	96.31	95.55	0.76	1.95	95.64	95.25	0.39	0.27
Proline	95.96	95.04	0.92	1.89	92.80	94.41	−1.61	0.84
Serine	89.87	89.11	0.76	1.22	90.64	91.35	−0.71	0.83
Tyrosine	96.86	96.73	0.13	1.03	94.32	94.24	0.08	0.64

^1^ PP25, PP:EGG ratio of 25:75; PP75, PP:EGG ratio of 75:25.

**Table 4:** Measured and predicted values for standardized ileal AA digestibility (%) in corn-gluten meal–egg mixtures using precision-fed cecectomized rooster assay.

	CGM25 ^1^				CGM75			
	Measured	Predicted	Difference	SEM	Measured	Predicted	Difference	SEM
Indispensable AA								
Arginine	96.22	95.79	0.43	0.73	93.02	92.89	0.13	2.11
Histidine	94.18	91.26	2.92 *	0.90	92.07	89.95	2.12	2.18
Isoleucine	96.69	96.40	0.29	0.60	93.38	92.62	0.76	1.47
Leucine	97.60	97.57	0.03	0.49	97.41	96.73	0.68	0.82
Lysine	94.75	93.62	1.13	0.72	85.65	86.65	−1.00	3.44
Methionine	97.82	97.50	0.32	0.48	96.19	95.47	0.72	1.02
Phenylalanine	97.06	96.85	0.21	0.56	95.20	94.38	0.82	1.25
Threonine	95.16	92.71	2.45	0.98	89.88	88.53	1.35	2.95
Tryptophan	99.84	99.92	−0.08	0.42	97.01	97.08	−0.07	0.58
Valine	96.38	95.91	0.47	0.67	93.40	92.55	0.85	4.25
Selected dispensable AA								
Alanine	96.62	96.42	0.20	0.44	96.08	95.34	0.74	1.10
Aspartic acid	94.12	92.28	1.84	0.82	90.01	89.13	0.88	1.79
Cysteine	91.56	88.62	2.94	1.55	85.45	80.83	4.62	3.04
Glutamic acid	95.47	95.18	0.29	0.70	95.84	94.46	1.38	0.92
Proline	94.42	93.91	0.51	0.89	94.63	92.61	2.02	1.43
Serine	90.92	88.29	2.63 *	0.94	90.22	88.86	1.36	2.25
Tyrosine	96.69	96.50	0.19	0.74	96.13	94.88	1.25	1.15

^1^ CGM25, CGM:EGG ratio 25:75; CGM75, CGM:EGG ratio of 75:25. * Labeled means in a row differ if measured compared with predicted *p* < 0.05.

## Data Availability

Data is unavailable due to privacy restrictions.

## Abbreviations

The following abbreviations are used in this manuscript:

AA	Amino acid
AAFCO	Association of American Feed Control Officials
CGM	Corn-gluten meal
CGM25	Mixture of CGM and EGG at a 25:75 ratio
CGM75	Mixture of CGM and EGG at a 75:25 ratio
CP	Crude protein
DIAAS	Digestible indispensable amino acid score
DM	Dry matter
FEDIAF	European Pet Food Industry Federation
NRC	National Research Council
EGG	Dehydrated egg
PP	Pea protein
PP25	Mixture of PP and EGG at a 25:75ratio
PP75	Mixture of PP and EGG at a 75:25 ratio
